# Mechanistic inferences on metabolic dysfunction in posttraumatic stress disorder from an integrated model and multiomic analysis: role of glucocorticoid receptor sensitivity

**DOI:** 10.1152/ajpendo.00065.2019

**Published:** 2019-07-19

**Authors:** Pramod R. Somvanshi, Synthia H. Mellon, Janine D. Flory, Duna Abu-Amara, PTSD Systems Biology Consortium;, Owen M. Wolkowitz, Rachel Yehuda, Marti Jett, Leroy Hood, Charles Marmar, Francis J. Doyle

**Affiliations:** ^1^Harvard John A. Paulson School of Engineering and Applied Sciences, Harvard University, Cambridge, Massachusetts; ^2^Department of Obstetrics, Gynecology & Reproductive Sciences, University of California, San Francisco, California; ^3^Department of Psychiatry, James J. Peters Veterans Affairs Medical Center, Bronx, New York; ^4^Department of Psychiatry, Icahn School of Medicine at Mount Sinai, New York, New York; ^5^Department of Psychiatry, New York Langone Medical School, New York, New York; ^6^Department of Psychiatry, University of California, San Francisco, California; ^7^Integrative Systems Biology, US Army Medical Research and Materiel Command, US Army Center for Environmental Health Research, Fort Detrick, Frederick, Maryland; ^8^Institute for Systems Biology, Seattle, Washington

**Keywords:** glucocorticoid signaling, HPA axis, mathematical modeling, neuroendocrine, PTSD

## Abstract

Posttraumatic stress disorder (PTSD) is associated with neuroendocrine alterations and metabolic abnormalities; however, how metabolism is affected by neuroendocrine disturbances is unclear. The data from combat-exposed veterans with PTSD show increased glycolysis to lactate flux, reduced TCA cycle flux, impaired amino acid and lipid metabolism, insulin resistance, inflammation, and hypersensitive hypothalamic-pituitary-adrenal (HPA) axis. To analyze whether the co-occurrence of multiple metabolic abnormalities is independent or arises from an underlying regulatory defect, we employed a systems biological approach using an integrated mathematical model and multiomic analysis. The models for hepatic metabolism, HPA axis, inflammation, and regulatory signaling were integrated to perform metabolic control analysis (MCA) with respect to the observations from our clinical data. We combined the metabolomics, neuroendocrine, clinical laboratory, and cytokine data from combat-exposed veterans with and without PTSD to characterize the differences in regulatory effects. MCA revealed mechanistic association of the HPA axis and inflammation with metabolic dysfunction consistent with PTSD. This was supported by the data using correlational and causal analysis that revealed significant associations between cortisol suppression, high-sensitivity C-reactive protein, homeostatic model assessment of insulin resistance, γ-glutamyltransferase, hypoxanthine, and several metabolites. Causal mediation analysis indicates that the effects of enhanced glucocorticoid receptor sensitivity (GRS) on glycolytic pathway, gluconeogenic and branched-chain amino acids, triglycerides, and hepatic function are jointly mediated by inflammation, insulin resistance, oxidative stress, and energy deficit. Our analysis suggests that the interventions to normalize GRS and inflammation may help to manage features of metabolic dysfunction in PTSD.

## INTRODUCTION

Posttraumatic stress disorder (PTSD) is defined by a complex set of criteria including intrusive reminders, fear memories, emotional distress, hypervigilance, and exaggerated startle responses that develop and persist after an exposure to trauma (1). Multiple physiological systems at the neuronal, metabolic, inflammatory, genomic, and epigenomic levels are known to be affected in PTSD (27, 69). Therefore, to understand the pathophysiology underlying the psychosomatic comorbidities, it is essential to study PTSD from a systems biological perspective (13). Previous studies from our PTSD Systems Biology Consortium reported associations between PTSD and insulin resistance (7), inflammation (44), reduced mitochondrial copy number (4), and lower methylation of the glucocorticoid receptor (*NR3C1*) gene (89) in the same cohorts assessed in the present study. The data from animal models and humans with PTSD reveal association between inflammation and metabolic syndrome (51). Analysis of metabolomics has revealed metabolic changes consistent with dysregulation in mitochondrial functioning in PTSD (50, 63). Another study reported differences in the mitochondrial DNA (single-nucleotide polymorphisms) located in NADH dehydrogenase and ATP synthase genes in PTSD (24). Variants of mitochondrial genes and dysregulation of their associated networks have been reported in the postmortem brains of patients with PTSD (77).

One of the major physiological regulatory axes, hypothalamic-pituitary-adrenal (HPA) axis, is implicated in the pathogenesis of PTSD and associated metabolic disorders (48, 87). The HPA axis relays stress signals from the brain to peripheral parts through the release of glucocorticoids (GCs) and catecholamine hormones. GCs orchestrate the activities of several physiological functions through glucocorticoid receptor (GR) signaling. Therefore, the feedback sensitivity of GC signaling, among other factors, can influence downstream effects of stress exposure. GR signaling is regulated at transcriptional and epigenetic levels and is dysregulated in HPA-axis-associated disorders (47). Several molecular processes may contribute to changes in GR sensitivity, such as intracellular hormone availability, GR expression levels, receptor isoform expression, conformational changes due to heat shock protein complex, hormone binding affinity, GR phosphorylation, and nuclear translocation (57, 58). Recent studies have highlighted the association of genetic polymorphisms of the GC receptor gene with metabolic disorders and PTSD (85). The role of GCs and GC receptor functioning in individuals with PTSD has been previously characterized (88); however, the underlying mechanisms by which the abnormalities in GR signaling contribute to metabolic abnormalities are not well delineated. Therefore, in light of the complexity of GC’s downstream regulatory effects, a systems-level analysis is required to understand dysregulation of the HPA axis and its metabolic consequences.

**Fig. 1.**
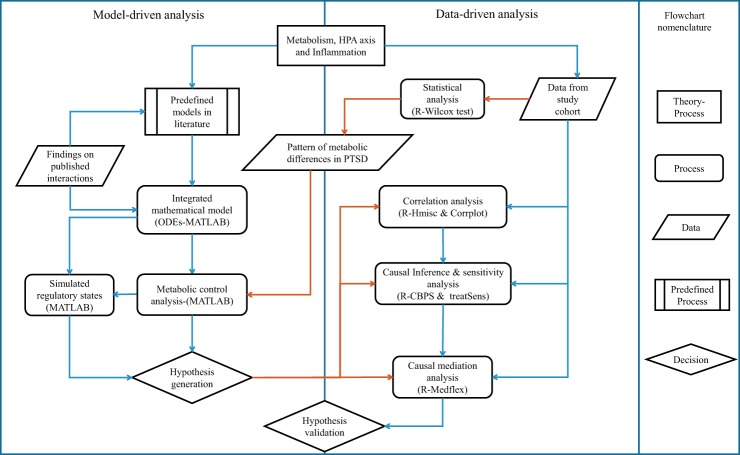
Process flowchart representing an outline of the steps that were implemented in the analysis presented in the paper. Published mathematical models for metabolism, hypothalamic-pituitary-adrenal (HPA) axis, inflammation, and hypoxia signaling were integrated into a single composite model based on the data reported in literature. Model was recalibrated, and metabolic control analysis (MCA) was performed to obtain metabolite concentration response coefficients (MCRCs) and associated regulatory states with reference to the pattern of statistically different metabolites in subjects with posttraumatic stress disorder (PTSD). Orange arrows represent flow of data from statistics to model analysis and causal inference. Response coefficients were used to generate hypothesis on the potential process that could be affected in PTSD. To validate the hypotheses, correlation analysis followed by causal inference and mediation analysis were performed using estimation of propensity scores and average causal effects, sensitivity analysis, and natural effects models. CBPS, covariate balancing propensity scores; ODEs, ordinary differential equations.

In view of these findings, we investigated whether the co-occurrence of the multiple metabolic abnormalities is independent or arises from a common underlying regulatory defect and its association with neuroendocrine changes observed in PTSD. To address this question, we used an integrative systems biological approach to identify regulatory connections in metabolic dysfunction in PTSD (72). The overall analysis is divided into two sections, namely model-based inferences: hypothesis generation using metabolic control analysis (MCA; Ref. 12) and data-based verification of the hypothesis using causal inference (33). The pipeline used for the current analysis is depicted in Fig. 1.

First, we performed statistical analyses on the data obtained from 83 combat-exposed veterans who developed PTSD and 82 combat-exposed veterans without PTSD to identify the significantly different metabolites and inflammatory and neuroendocrine features in their plasma samples. The demographics of our sample are reported in Supplemental Table S1 (all supplemental material is available at https://doi.org/10.5281/zenodo.3344984). We used the pattern of change in significantly different metabolic pathway components as the reference for MCA. We refer to the pattern of change in these metabolites as metabolic dysfunction (MD) signature. Second, we integrated the ordinary differential equations (ODEs)-based mathematical models from the literature for metabolism, HPA-axis activity, and inflammation to identify the putative mechanisms underlying this metabolic signature in combat-related PTSD. With the use of this model, MCA was conducted to determine potential perturbations in the model that could yield consistent metabolic differences as observed in our PTSD sample. The parameters associated with these perturbations were used to generate hypotheses on the processes that could potentially be affected in PTSD. Specifically, we determined the parameters representing dysregulation in HPA axis, GR signaling, G protein-coupled receptor (GPCR) signaling, and inflammatory pathways that could reproduce the MD signature as observed in our subjects with PTSD. Among these mechanisms, GR sensitivity of HPA-immune axis showed higher control on the metabolic differences. Therefore, we focus our further analysis on the effects of enhanced GR sensitivity on metabolism. Although this paper focuses on the plausible effects of enhanced GR sensitivity on metabolism, the precise mechanism by which GC/GR sensitivity is changed in PTSD is outside of the scope of the present study.

To ascertain the MCA-based inferences on the underlying mechanisms for the observed metabolic differences using our data, we performed correlational analysis and causal inference (86). We incorporated the results from neuroendocrine, cytokine, and clinical laboratory assays along with significantly different metabolites for our subjects in these analysis. The analyses supported the MCA-based findings with statistically significant associations and average causal effects between cortisol suppression (glucocorticoid receptor sensitivity), high-sensitivity C-reactive protein (hs-CRP; inflammation), homeostatic model assessment of insulin resistance (HOMA-IR), γ-glutamyltransferase (GGT; oxidative stress), hypoxanthine (energy deficit and hypoxia), and several metabolites. Furthermore, the estimates from average causal effects were used to inform the causal mediation hypothesis that was tested using natural effects models with inverse probability weighting (75). We conducted causal mediation analysis (CMA) with cortisol suppression (CS) by dexamethasone (DEX) as an exposure variable, HOMA-IR, hs-CRP, hypoxanthine, and GGT as the joint mediators, and metabolites as dependent variables while controlling for covariates (80). CMA suggests that the mechanistic association of enhanced glucocorticoid receptor sensitivity with metabolic dysfunction is partly mediated by inflammation, oxidative stress, insulin resistance, and energy deficit.

The mechanistic inferences from our analysis imply that the interventions to normalize glucocorticoid sensitivity may help to manage features of metabolic dysfunction in PTSD. Alternatively, targeting inflammation, oxidative stress, and mitochondrial metabolism can restore energy deficit, thereby normalizing the metabolic abnormalities in PTSD. Our analysis provides a basis for future prospective experiments on animal models to test the therapeutic potential of these targets and develop treatment modalities to manage somatic comorbidities in PTSD.

## MATERIALS AND METHODS

### Participants

The data from 165 men between 20 and 60 yr of age who served in Iraq (Operation Iraqi Freedom, OIF) or Afghanistan (Operation Enduring Freedom, OEF) and participated in the study as part of a systems biology approach to identify biomarkers for PTSD in OEF/OIF veterans were included in the analyses for this paper. Participants were recruited at two sites including the James J. Peters Veterans Affairs Medical Center (JJPVAMC)/Icahn School of Medicine at Mount Sinai (ISMMS) and New York University Langone Medical Center (NYULMC)/NYU School of Medicine through advertising in the clinic (VAMC) and community (local colleges and universities, veterans’ centers, and media advertisements). All participants provided written, informed consent for study procedures, and the study was approved by the institutional review boards of the JJPVAMC, ISMMS, and NYULMC and the Human Research Protection Office at the United States Army Medical Research and Materiel Command.

### Clinical Assessments and Biochemical Assays

The details on clinical assessment procedures for characterization of patients with PTSD and diagnostic criteria, blood and urine sample collection, clinical laboratory and cytokine assays, neuroendocrine assays for characterization of HPA axis by cortisol and ACTH, cortisol and ACTH suppression by dexamethasone suppression test, lysozyme IC_50_-DEX, dehydroepiandrosterone, urine catecholamines (epinephrine, norepinephrine, and dopamine), and metabolomics assays are reported in data supplements. The original data used in this study can be referred from another publication of ours (15a).

### Statistical Analysis

The data for metabolomics, neuroendocrine, clinical laboratories, and cytokines were log-transformed and median-normalized before the statistical analysis. We used nonparametric Mann–Whitney *U* test to identify the features that show statistically significant difference in the PTSD and control groups. The statistical significance for all analysis was set at α = 0.05, and trend-level significance was set to 0.05 < α < 0.1. The false discovery rate and *q* values for the multiple comparisons were obtained using qvalue package in R. R package Hmisc was used to obtain the Spearman correlation coefficients (ρ) and the R package corrplot (84) to plot correlation plots.

### Mathematical Model Development

To analyze the defects in metabolism and its regulation, we integrated the metabolic simulator with the models for HPA axis, inflammation, and hypoxia and simulated metabolic trends. We adapted four submodels published in literature, namely hepatic metabolism and its hormonal regulation (71), HPA axis and inflammation model (2, 60), glucocorticoid receptor model (65), and hypoxia signaling (56). These models were integrated by linking the regulatory nodes according to the interactions reported in the literature (Supplemental Table S3). The integrated model basically consists of four modules viz. HPA axis, inflammation pathway, metabolic network, and metabolic regulatory signaling and transcription network. These subsystems are considered in a modular fashion for the sake of regulatory integration between the modules. Although the model involves different organ systems (brain, adrenals, and liver), we are dealing with the variables in the plasma that are obtained as clinical features. Therefore, for the sake of simplicity, we have assumed homogeneously mixed concentrations of the plasma features that are also assumed to be in equilibrium with the native tissues. Hence, the features remain the same throughout the modules unless defined by a different name in two different tissue compartments (such as cytosol and mitochondria, brain and periphery).

The model comprises major metabolic pathways of glycolysis, TCA cycle, amino acid metabolism, urea cycle, lipid metabolism, and plasma metabolite transport (32) along with regulatory signaling pathways for insulin (insulin receptor substrate-Akt; Ref. 68), glucagon and catecholamine (GPCR-cAMP-PKA; Refs. 45, 54), calcium signaling (36), mammalian target of rapamycin (mTOR) signaling (81), HPA axis for cortisol and glucocorticoid receptor (GR) dynamics, inflammation, and hypoxia (Fig. 3, *A*–*C*). We used a semiempirical approach to incorporate regulatory effects by using the saturating rate equations modeled by Michalis–Menten and Hill type of biochemical kinetics. The equations were formulated to reproduce the qualitative trends in experimentally observed physiological responses at basal conditions and for changing the input stimulus. The overall model comprises 189 ODEs for the state variables. The model was validated to reproduce the qualitative physiological responses for different input conditions (Supplemental Fig. S1). The model was developed and analyzed using MATLAB (2017a). The details of the integrated model development and model equations are reported in the appendix. The model parameters are reported in Supplemental Tables S3 and S4.

The general scheme of model formulations implemented the methodology followed in Refs. 8, 43, and 71, wherein the concentrations of metabolic state variables were modeled by ODEs:

(1) $$\frac{{\text{dM}}_{i}}{\text{d}t}\times \text{V}=\sum_{j=1}^{nj}{\text{RP}}_{j}-\sum_{k=1}^{nk}{\text{RC}}_{k}\pm {\text{T}}_{\text{t}}\text{,}$$

where M*_i_* represents the *i*th metabolite; V represents the volume of the compartment to which the metabolite belongs; RP*_j_* and RC*_k_* are the rate of production and consumption of a metabolite, respectively; and T_t_ represents the transport of metabolite across blood and tissue. The rates of formation and consumption are further modeled as:

(2) $${\text{RP}}_{j}={\text{V}}_{{\text{max}}_{j}}\times F\_{\text{RP}}_{j}\times \overset{nfj}{\underset{f=1}{\prod }}\left(\frac{{\text{M}}_{f,j}}{{\text{M}}_{f,j}+K{\text{m}}_{f,j}}\right)$$

(3) $$\text{F\_}{\text{RP}}_{j}=\overset{nrj}{\underset{r=1}{\prod }}\left({\text{F}}_{{\text{Act}}_{r,j}}\times {\text{F}}_{{\text{Deact}}_{r,j}}\times {\text{F}}_{\text{pi}}\times {\text{F}}_{{\text{Sig}}_{{\text{trans}}_{r,j}}}\right)$$

(4) $${\text{F}}_{{\text{Act}}_{r,j}}=\left(\frac{\text{A}}{\text{A}+K_{j}}\right);\;{\text{F}}_{{\text{Deact}}_{r,j}}=\left(\frac{K_{i}}{\text{I}+K_{i}}\right);\;{\text{F}}_{\text{pi}}=\left[\frac{\text{A}}{\text{A}+K_{i}\times \left(1+\frac{\text{I}}{K_{p}}\right)}\right]$$

(5) $${\text{F}}_{{\text{Sig}}_{{\text{trans}}_{r,j}}}=Wf\times \left(1+\overset{an}{\underset{a}{\sum }}{\text{Reg}}_{s_{{\text{act}}_{a}}}+\overset{bn}{\underset{b}{\sum }}{\text{Reg}}_{t_{{\text{act}}_{b}}}\right)\times \overset{dn}{\underset{d}{\prod }}\left({\text{Reg}}_{s}\_{\text{deact}}_{d}\times {\text{Reg}}_{t}\_{\text{deact}}_{d}\right)$$

(6) $${\text{Reg}}_{st}\text{\_}{\text{act}}_{j}\overset{pn}{\underset{p}{\prod }}\left(\frac{{\text{S}}_{p}^{na}}{{\text{S}}_{p}^{na}+{\text{K}}_{p}^{na}}\right)$$

(7) $${\text{Reg}}_{s}\_{\text{deact}}_{j}=\overset{qn}{\underset{q}{\prod }}\left(\frac{K_{p}^{nd}}{I_{p}^{nd}+K_{p}^{nd}}\right)$$

(8) $${\text{T}}_{\text{t}j\text{passive}}={\text{ε}}_{j}\times \left({\text{C}}_{\text{b}j}-{\text{C}}_{\text{cyt}j}\right)$$

(9) $${\text{T}}_{\text{t}j\text{Faciltated}}={\text{T}}_{j}\times \left(\frac{{\text{C}}_{\text{b}j}}{K_{\text{b}j}+{\text{C}}_{\text{b}j}}-\frac{{\text{C}}_{\text{cyt}j}}{K_{\text{cyt}j}+{\text{C}}_{\text{cyt}j}}\right)\text{,}$$

where F_RP*_j_* is the regulatory function governing the rate of *j*th enzyme; M*_f_*_,_*_j_* is the *f*th metabolite in *j*th reaction; F_Act_*r*,*j*__, F_Deact_*r*,*j*__, and F_pi_ represent the activation rate, deactivation rate, and product inhibition rate by *r*th metabolite in the *j*th reaction; A and I represent the activator metabolite and inhibitor metabolite; ${\text{F}}_{{\text{Sigtrans}}_{r,j}}$ represents the regulation by a signaling or a transcription regulator for *r*th metabolite in the *j*th reaction; *Wf* is the weighting factor; Reg*_st_*_act*_j_* and Reg*_s_*_deact*_j_* are the activation rate and deactivation rate by an activator S and an inhibitor I with a sensitivity of *na* and *nd* and saturation constant *K*, respectively; ε represents the partition coefficient of a metabolite; and T_t_*_j_*_passive_ and T_t_*_j_*_Facilitated_ represent the passive and facilitated transports of *j*th metabolites to and from the tissues to blood, respectively, where C_b_*_j_* and C_cyt_*_j_* are the concentrations of the metabolite in blood and tissue, respectively.

### Metabolic Control Analysis

Metabolic control analysis (23, 73) was performed by perturbation of parameters in the model, wherein parameters represent rate of reactions and strength of interaction in the metabolic and regulatory network, respectively. We performed the metabolic control analysis to obtain concentration response coefficients to identify the rate parameters that can yield the metabolic signatures as showed by statistically significant group differences in subjects with and without PTSD. To quantify the changes in metabolite concentrations with respect to changes in the parameters for reactions or regulatory interactions, we calculate metabolite concentration response coefficients (MCRCs) as:

(10) $$\text{MCRC}=\left(\frac{\text{dC}}{\text{d}p}\times \frac{\text{P}}{\text{C}}\right)\text{,}$$

where C and P are the concentration of a metabolite and parameters for reaction rates in the model, respectively. We recorded MCRCs for 100 metabolic reaction rates and 260 signaling and transcriptional regulatory interaction rates, with respect to the trends observed in 12 metabolites that were identified significantly different in PTSD data set. Because of the nonlinear nature of the regulatory influences of hormonal signaling and transcriptional factors, instead of infinitesimal perturbation, we obtained the response coefficients for a modest perturbation across 50% change in the reaction and interaction rates (i.e., 1.5- and 0.5-fold of the native value). We obtained the net response coefficient by taking the mean of response coefficients recorded for decreasing (0.5-fold) and increasing (1.5-fold) the parameter across the nominal values and screened the mean cumulative MCRCs with at least MCRC of 0.001 for each of the 12 metabolites. The steady-state simulation results for those 12 metabolites were compared with the pattern of change in direction of significantly different metabolites in subjects with PTSD. The directions of the change in metabolite concentrations (positive or negative) with respect to baseline levels for a particular rate perturbation were used to determine whether a concentration of a metabolite would decrease or increase for a perturbation in a rate of reaction. The control coefficients that yielded all of the changes as observed in the MD signature were recorded for further inferences related to the disease. The simulations for MCA were carried out under constant glycogen levels to mimic the normal supply of energy source and maintain steady-state levels in the model.

### Average Causal Effects

We used the covariate balancing propensity scores (CBPS; Ref. 16) package in R to obtain the weights for covariate/confounder adjustments while deducing causal effects. The covariate balancing generalized propensity scores obtained from CBPS are optimized to maximize both the covariate balance and prediction of treatment assignment in the sample by minimizing the association between covariates and the treatment/exposure variable. A nonparametric version called npCBPS was used to obtain the weights for fitting the average causal effects (ACEs). To obtain the population-level causal estimates, we used the entire sample for analysis by controlling for the group effects. We computed the average causal effects (ACE-γ) of 6 regulatory components using the CBPS package, with respect to 35 metabolites in our sample. The ACEs [represented by gamma (γ)] can be interpreted as the percentage change in the affected metabolite per unit percentage change in the regulatory component. ACEs capture the average causal association between two variables controlling for covariates without referring to the direction of causality.

### Sensitivity Analysis of Causal Estimates

We used treatSens package in R (11) to perform sensitivity analysis (SA) of the causal estimates for unmeasured confounding. The SA employs a simulation-based nonparametric method, wherein a sensitivity parameter is the coefficient of the association between the unknown confounder and the treatment and outcome variables. The method determines the sensitivity estimates that can account for the bias due to model misspecification and unmeasured confounding. The data were standardized for the SA to obtain the sensitivity coefficient ranging from 0 to 1. The sensitivity is assessed graphically by plotting the two-dimensional plot of the causal estimates with respect to biparametric variation (correlation of unknown confounder with treatment and outcome variables; Ref. 20). We recorded sensitivity parameters representing the intersection of the biparametric curve with the *x* = *y* line on the plot. Two sensitivity parameters were recorded, namely, τ_1_, the point of intersection of the *x* = *y* line with the curve that represents the causal estimate equals to 0, and τ_2_, the point of intersection of the *x* = *y* line with the curve that represents the lack of significance (where *P* > 0.05) of the causal estimate. The sensitivity parameters were evaluated only for the statistically significant causal associations.

### Causal Mediation Analysis

We used the medflex package in R (76) to perform causal mediation analysis using natural effect models (75). The package employs computations on conditional mean models for nested counterfactuals to estimate the causal effects. These models allow for estimation of natural direct and indirect or mediated effects (NDE and NIE) through its coefficients, which provides easier interpretation of effects with respect to the exposure variables. The causal mediation analysis uses a counterfactual framework of causal inference that works by identifying the difference between the potential outcomes of a design or a graph with and without a mediator estimated by the following equations:

(11) $$\text{NDE}\left(0\right)=\text{E}\left\{Y\left[T=T1,M\left(0\right)\right]-Y\left[T=0,M\left(0\right)\right]\right\}$$

(12) $$\text{NIE}\left(1\right)=\text{E}\left\{Y\left[T=T1,M\left(1\right)\right]-Y\left[T=T1,M\left(0\right)\right]\right\},$$

where E stands for expectation and *Y*, *T*, and *M* are the outcome variable, treatment or exposure variable, and mediator variable, respectively. The suffixes 1 and 0 in parentheses represent the notation for with and without the change in treatment or mediator variables. To obtain generalized estimates of the causal effects, we performed a population-level mediation analysis on the entire cohort controlling for the disease status. The population-level effects were thus obtained by weighting by the reciprocal of the conditional exposure density of the treatment variable through inverse probability weighting in the functional models. The causal inference problem is based on assumptions of sequential ignorability on the causal structure invoked by nonparametric structural equation models with independent error terms (61). NDE (ψ*_d_*) is interpreted as, for a subject with baseline covariates, 1% change from average level of cortisol suppression results in a percentage change in a corresponding metabolite level by the factor of NDE. NIE (ψ*_i_*) can be interpreted as for altering the level from that which would have been observed at the average levels of cortisol suppression to the level that would have been observed at 1% change in cortisol suppression, which would result in the percentage change in metabolite level by the factor of NIE. The total causal effect (ψ*_t_*) is the sum of the direct and indirect effects.

## RESULTS

### Group Differences in Features Between PTSD and Controls

**Fig. 2.**
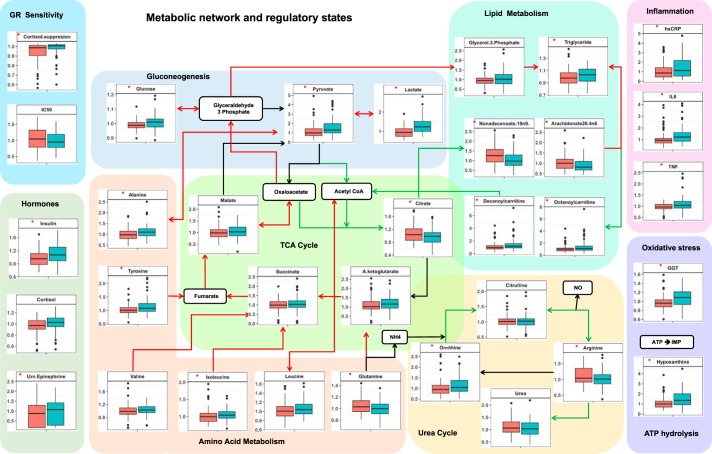
Box plot representation of metabolite differences in controls and posttraumatic stress disorder (PTSD). Red arrows represent the upregulated fluxes, and green arrows represent downregulated metabolic fluxes. Side panels represent differences in metabolic regulatory hormones, tests for glucocorticoid receptor (GR) sensitivity, and markers for inflammation, oxidative stress, and ATP hydrolysis. Red asterisks on the box plots represent the statistically significant difference (*P* < 0.05) in PTSD vs. controls. Green box, PTSD; red box, controls. It is noted that gluconeogenesis potentially in liver, hypoxic adaptation potentially in muscle, amino acid catabolism, triglyceride synthesis, and ATP hydrolysis are upregulated, whereas urea cycle, lipogenesis, and β-oxidation are downregulated. Measures of GR sensitivity, inflammation, and oxidative stress and the hormones insulin, cortisol, and urinary (Urn.) epinephrine are also elevated in PTSD. GGT, γ-glutamyltransferase; hsCRP, high-sensitivity C-reactive protein.

To assess the group differences in our data, we performed a nonparametric Mann–Whitney *U* test followed by adjustments for false discovery rate and estimation of effect sizes given by Cohen’s *d*. The summary statistics for group differences in metabolites that showed *P* value <0.05 and *q* value <0.1 are reported in Supplemental Table S2. We identified 31 metabolites, 2 cytokines, 14 clinical variables, and 3 neuroendocrine variables significantly different (at *P* value <0.05 and *q* value <0.1) between PTSD and control subjects. The alterations in the metabolite flows in the metabolic pathways are depicted in Fig. 2. These findings can be attributable to mitochondrial dysfunction (50) inferred by the disturbance in the inflow-outflow of metabolites (carbohydrates, fatty acids, and amino acids) and mitochondrial metabolite processing (TCA cycle, β-oxidation, urea cycle, and amino acid catabolism).

### Metabolic Control Analysis for Metabolic Signature in PTSD

**Fig. 3.**
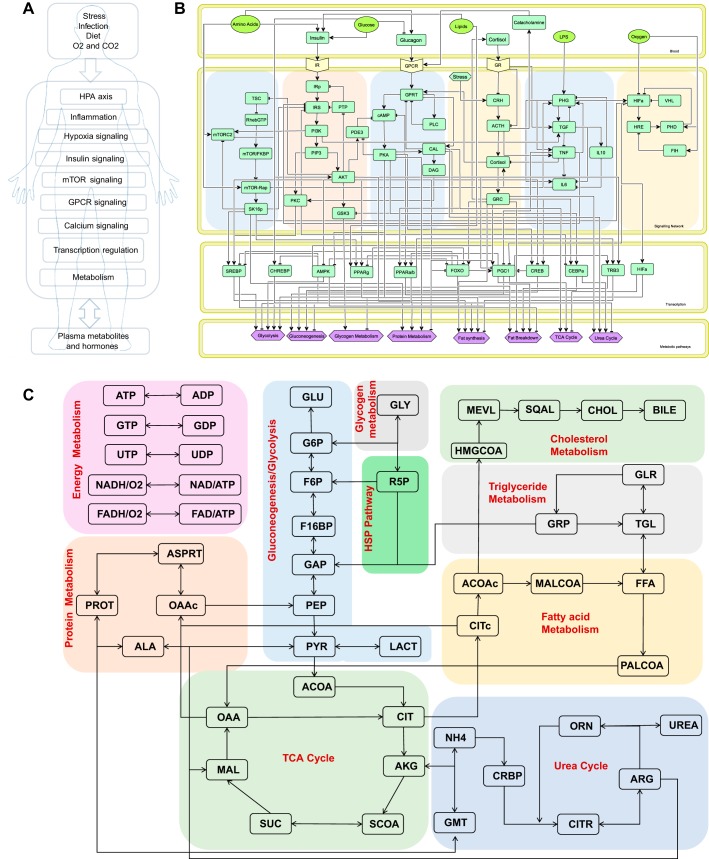
*A*: schematic of the components of the integrated model. *B*: regulatory network representing 6 signaling pathways, 10 transcription factors, and the metabolic processes that are regulated by the network. Network comprises *1*) mammalian target of rapamycin (mTOR) pathway, *2*) insulin signaling pathway, *3*) G protein-coupled receptor (GPCR) signaling pathway, *4*) hypothalamic-pituitary-adrenal (HPA) axis, *5*) inflammatory signaling, and *6*) hypoxia signaling. These signaling pathways interact to activate downstream transcription regulatory network as shown in the transcription factor compartment. Signaling and transcription network collectively influence metabolic processes as represented in the metabolic pathways compartment. *C*: metabolic pathways coded in the metabolic simulator. Model constitutes glycolysis, gluconeogenesis, TCA cycle, urea cycle, lipogenesis, amino acid metabolism, hexose amine pathway, pentose phosphate pathway, oxidative phosphorylation, cholesterol pathway, and plasma metabolite fluxes. This model is further integrated with regulations from the regulatory network. ACOAc, acetyl Co-enzyme A (cytosolic); ADP, adenosine diphosphate; AKG, alpha-ketoglutarate; ALA, alanine; ARG, arginine; ASPRT, aspartate; ATP, adenosine triphosphate; CHOL, cholesterol; CIT, citrate; CITc, citrate (cytosolic); CITR, citrulline; CRBP, carbamoyl phosphate; F16BP, fructose-1-6-biphosphate; F6P, fructose-6-phosphate; FAD, flavin adenine dineucleotide; FFA, free fatty acids; GAP, glyceraldehyde phosphate; GDP, guanosine diphosphate; GLR, glycerol; TGL, triglycerides; GLU, glucose; G6P, glucose-6-phosphate; GLY, glycogen; GMT, glutamine; GRP, glycerol-3-phosphate; GTP, guanosine triphosphate; HMGCOA, β-hydroxy-β-methylglutaryl-CoA; LACT, lactate; MAL, malate; MALCOA, malonyl co-enzyme A; MEVL, mevelonate; NAD, nicotinamide adenine dinucleotide; NADP, nicotinamide denine dinucleotide phosphate; NH4, ammonia; OAA, oxaloacetic acid; OAAc, oxaloacetic acid (cytosolic); ORN, ornithine; PALCOA, palmitoyl co-enzyme A; PEP, phopsphoenoyl pyruvate; PROT, protein; PYR, pyruvate; R5P, ribose-5-phosphate; SCOA, succinyl Co-enzyme A; SQAL, squalene; SUC, succinate; UDP, uridine diphosphate; UTP, uridine triphosphate.

**Fig. 4.**
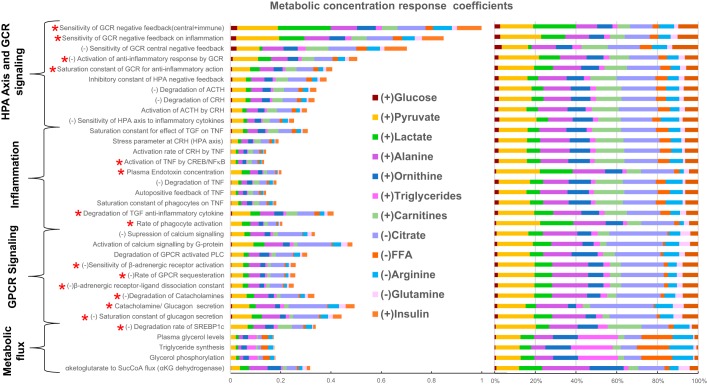
Plot represents the result of metabolic control analysis: 34 model parameters that elicited the metabolic signature as observed in subjects with posttraumatic stress disorder along with their mean cumulative metabolic concentration response coefficient measured across 12 metabolites with ≥0.1 mean cumulative metabolite concentration response coefficient. Sign (±) in prefix of the parameters represents the direction of change that yielded the metabolic dysfunction signature. These parameters belong to the hypothalamic-pituitary-adrenal (HPA)-axis and glucocorticoid receptor (GCR) signaling, G protein-coupled receptor (GPCR) signaling, inflammation, and metabolic fluxes for triglyceride synthesis, plasma lactate, and amino acid levels (Supplemental Table S5). Red asterisk at the prefix of the labels indicates the parameters that were selected when additionally accounted for group differences in cortisol, ACTH, IL6, and TNF along with the 12 metabolites. Colors in the bars code for the fraction of the response coefficients corresponding to each metabolite. It is noted that citrate and pyruvate were mostly affected by these parameters contributing to ~40% of the total effect per parameter followed by the effect on amino acid (alanine and glutamine) concentration (~15%), lipid metabolite (carnitine, fatty acids, and triglycerides) concentration (~15%), urea cycle metabolites (~15%), and glucose, lactate, and insulin together (~15%). CREB, cAMP response element-binding; CRH, corticotropin-releasing hormone; FFA, free fatty acid; SREBP1c, sterol regulatory element-binding protein 1c; TGF, transforming growth factor.

To analyze the putative mechanisms underlying the observed changes in metabolism, we used a systems-level mathematical model composed of hepatic metabolism integrated with mathematical models for the metabolic regulatory signaling and transcription, HPA-axis and GR signaling, and inflammation and hypoxia signaling from the literature (Fig. 3). With the use of the model, MCA was conducted to determine potential perturbations in the model that could yield a consistent MD signature. Through MCA, we obtained metabolite concentration response coefficients (MCRCs) with respect to the trends in the significantly different metabolic pathway components represented by 12 metabolites, namely glucose, pyruvate, lactate, citrate, alanine, glutamine, long-chain fatty acids, triglycerides, carnitines, arginine, ornithine, and insulin (Supplemental Table S2: subsections represent pathways). MCRC quantifies the degree of control exerted by the change in a parameter value on the concentration of a metabolite. We estimated MCRCs for 360 model parameters and identified 34 rate parameters that could yield a mean cumulative response coefficient of ≥0.1 for 12 metabolites taken together (with MCRC of ≥0.001 for each metabolite), across 50% perturbation in the parameter. Figure 4 shows the mean cumulative control coefficients of parameters with relative contribution toward each metabolite perturbation. These parameters, if perturbed, yielded a consistent MD signature, in terms of the direction of change (whether increased or decreased) in subjects with PTSD with respect to controls. Among the most influential parameters, those increasing systemic glucocorticoid receptor sensitivity (GR sensitivity of HPA-axis central negative feedback and sensitivity of cytokine regulation taken together: parameter *n* and *nx* in model equations) yielded the highest control coefficient, followed by parameters for perturbations in inflammatory response and GPCR pathway. Although individually increasing the sensitivity of GR’s anti-inflammatory effect and central negative feedback effect produced opposite effects, the model yielded the MD signature on simultaneously increasing GR sensitivity in these pathways indicating a synergistic effect of the GR hypersensitivity of HPA axis and immune system on metabolism. The changes in either molecular process that determines GR sensitivity affect the steepness of the dose-response curve of the GR activity that is denoted by Hill coefficient of GR effects in the mathematical model. The rate parameters and corresponding processes along with the mean cumulative MCRCs (MCRC of ≥0.001 for each the 12 metabolites) are summarized in Supplemental Table S5.

### Model-Based Inference from Simulated Regulatory States

**Fig. 5.**
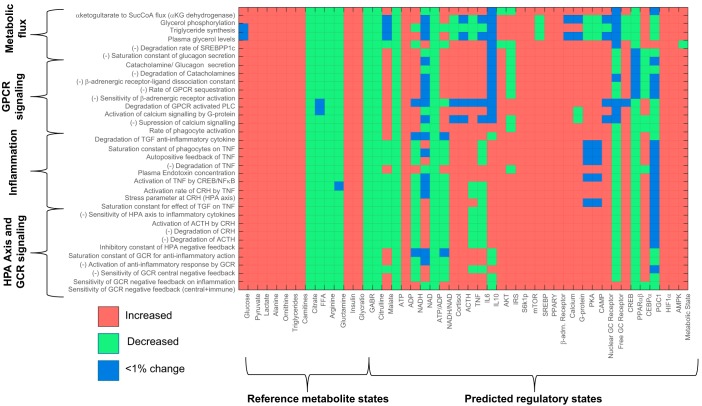
Matrix representation of the changes in the states of metabolite and regulatory component with respect to reference state for perturbation in the 34 parameter, identified through metabolic control analysis. States are coded as either increased (red) or decreased (green) and <1% change (blue) per cell corresponding to the parameter-state combination. It can be noted that the trends in reference metabolites (metabolic dysfunction signature observed in posttraumatic stress disorder) are replicated for the parameters on *y*-axis. Predicted states of other regulatory components corresponding to these parameter perturbations are shown appended to reference metabolites. It is noted that G protein-coupled receptor (GPCR) pathway and glucocorticoid (GC) receptor (GCR) nuclear translocation are upregulated for most parameters along with an upregulation of catabolic state. ATP-to-ADP ratio is also reduced for all of the parameter perturbation along with upregulation of cAMP response element-binding (CREB), AMP-activated protein kinase (AMPK), and hypoxia-inducible factor 1α (HIF1α). Net catabolic state can be observed (last column: metabolic state) for all of the 34 parameter perturbations. β-adrn., β-Adrenergic; CEBPα, CCAAT/enhancer-binding protein-α; CRH, corticotropin-releasing hormone; FFA, free fatty acid; GABR (global arginine bioavailability ratio), arginine/(ornithine + citrulline); Glycratio (glycolytic ratio), (pyruvate + lactate)/citrate; HPA, hypothalamic-pituitary-adrenal; IRS, insulin receptor substrate; mTOR, mammalian target of rapamycin; PGC1, peroxisome proliferator-activated receptor-γ coactivator 1; PPARγ, peroxisome proliferator-activated receptor-γ; S6k1p, phosphorylated ribosomal protein S6 kinase; SREBP1c, sterol regulatory element-binding protein 1c; TGF, transforming growth factor.

To probe the mechanisms by which the MD signature was achieved for these 34 parameter perturbations, we further analyzed the corresponding metabolic regulatory states for each parameter perturbation through the model simulations. We recorded the states of regulatory components such as ATP-to-ADP and NADH-to-NAD ratios, anabolic and catabolic signaling pathway components, transcriptional factors, and inflammatory pathway as shown in Fig. 5. It was consistently noted that metabolic controller ratio ATP to ADP was reduced for all of the 34 parameter perturbation cases. The oxidation-reduction (redox) ratio NADH to NAD was elevated for upregulation of GPCR signaling, whereas it was reduced for the parameters for HPA-axis activation-associated inflammatory state. In the insulin signaling pathway, Akt phosphorylation was increased relative to the basal level except for the parameters of metabolic flux category, with reduced insulin receptor substrate tyrosine phosphorylation for GPCR parameters. The components of amino acid metabolic regulators, mTOR pathway (mTOR raptor and S6K1p), and transcriptional regulators of lipid synthesis pathway [sterol regulatory element-binding protein (SREBP) and peroxisome proliferator-activated receptor-γ (PPARγ)] were also activated for perturbation in all of the parameters of the regulatory category. Among the components of GPCR pathway, β-adrenergic receptor, G protein, and calcium signaling were activated for all of the parameters along with the upregulation of protein kinase A (PKA) and cyclic AMP (cAMP).

The global transcriptional regulator cAMP response element-binding (CREB) was activated across all parameters. The transcriptional regulator for lipid catabolism PPARα/β was downregulated throughout the perturbations in the parameters of HPA axis and inflammation and was upregulated for GPCR signaling. The transcriptional regulator for urea cycle, CCAAT/enhancer-binding protein-α (CEBPα), was upregulated only for the parameters in the inflammation and HPA-axis classes and downregulated for GPCR parameters. The transcriptional regulator for mitochondrial biogenesis, peroxisome proliferator-activated receptor-γ coactivator 1α (PGC1α), was also marginally downregulated for the parameter perturbations in glucocorticoid receptor sensitivity and inflammatory signaling but remained unchanged for the parameters in GPCR and other HPA-axis signaling. The hypoxia-inducible factor HIF1α and AMP-activated protein kinase (AMPK) were upregulated throughout the perturbations in the parameters of all four categories. The nuclear translocation of glucocorticoid receptor was enhanced for the parameters of the HPA-axis and inflammatory signaling, with increased levels of cortisol and ACTH. The levels of inflammatory cytokines TNF and IL6 were upregulated for the parameters of inflammatory signaling and downregulated for the HPA-axis perturbations except for increase glucocorticoid sensitivity. The metabolic state was catabolic across all of the parameters listed except for elevated SREBP1c expression and plasma amino acid flux.

### MCA-Based Hypothesis for Metabolic Dysfunction in PTSD

On further accounting for the increase in the inflammatory cytokines (IL6 and TNF) and the HPA-axis variables (ACTH and cortisol) along with the 12 metabolic components, 16 out of 34 parameters (indicated by red asterisks in Fig. 4) constituting GR’s anti-inflammatory effect, inflammation pathway, and GPCR signaling produced a response consistent with MD signature comprising these 16 features. From the MCA and the analysis of associated regulatory states, it can be inferred that the steady-state perturbation in the parameters for HPA-axis and β-adrenergic signaling are accompanied by the combination of changes in metabolic controller ratios (ATP to ADP and NADH to NAD), inflammatory response, insulin resistance, and catabolic state associated with an overall energy deficit. Hence, we hypothesize that the trauma-induced changes in the systemic glucocorticoid receptor sensitivity could be mechanistically associated with insulin resistance, inflammation, oxidative stress, energy deficit, and subsequent metabolic dysfunction in PTSD.

### Correlational Analysis

**Fig. 6.**
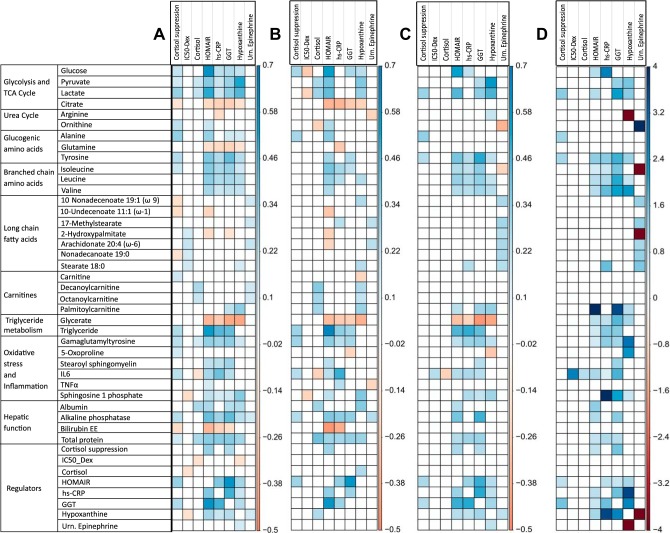
Correlation plot for regulatory components with respect to metabolites in entire cohort (*A*), controls (*B*), subjects with posttraumatic stress disorder (PTSD; *C*), and fold change in correlations with respect to correlations in controls (the fold changes >4 are capped at 4; *D*). Correlations are shown for the statistically significant correlations (*P* < 0.05; Supplemental Table S6). It is observed that across the correlation plots *A*–*C*, significant correlations are conserved for the cross-correlations between homeostatic model assessment of insulin resistance (HOMAIR), γ-glutamyltransferase (GGT), high-sensitivity C-reactive protein (hs-CRP), hypoxanthine and glycolytic metabolites, amino acid metabolites, and triglyceride metabolites. On the fold change plot, it is noted around 2- to 4-fold higher correlations in subjects with PTSD between GGT, hs-CRP, hypoxanthine, and HOMAIR, indicating stronger association between oxidative stress, inflammation, and insulin resistance, along with similar level of increase in correlation between regulatory components and metabolites except for fatty acids. There is a notable increase in association between cortisol suppression and citrate, alanine, tyrosine, HOMAIR, and GGT. Dex, dexamethasone; Urn., urinary.

To ascertain the MCA-based findings in our data, we obtained Spearman correlation coefficients between 8 regulatory components and 35 metabolites for controls and PTSD and compared the fold changes in associations across the cohorts (see Fig. 6 and Supplemental Table S6*A*). We used cortisol suppression [a measure of GC feedback sensitivity in the HPA axis (39): it is assumed that the extent of cortisol suppression or ACTH suppression by dexamethasone (DEX) administration is proportionate to increase in GR sensitivity], IC_50_ of lysozyme activity in response to DEX treatment (concentration of DEX at which 50% of lysozyme activity was inhibited), cortisol, urinary epinephrine (activator of β-adrenergic-GPCR pathway), hs-CRP (a marker for inflammation), homeostatic model assessment of insulin resistance (HOMA-IR), hypoxanthine (a surrogate for hypoxia and energy deficit; Refs. 30, 66), and γ-glutamyltransferase (GGT; a surrogate for oxidative stress; Ref. 34) as the regulatory components that represented variables highlighted by MCA. The selection of metabolites for this analysis was made based on the statistical significance and the relevance in context of the model-based hypothesis. Because of their associations with the metabolic pathways that showed statistically significant differences, despite the trend-level significance between groups in citrate, isoleucine, glycerate, carnitine and palmitoylcarnitines, branched-chain amino acids (leucine and valine), and arginine, these metabolites were included in the correlational analysis (features suffixed by asterisk in Supplemental Table S2). Since the essential fatty acids need to be absorbed from the food and are not likely to be associated with the perturbations in metabolic pathways, we excluded them from this analysis.

Cortisol suppression by DEX strongly correlated with HOMA-IR, GGT, and several other metabolites (Fig. 6, *A*–*C*). It is noted that the cross-correlations for hs-CRP, GGT, hypoxanthine, HOMA-IR, and the metabolites were elevated by two- to fourfold in PTSD with respect to controls (Fig. 6*D*). The correlation coefficients for the entire cohort and subjects with PTSD are reported in Supplemental Table S6, *A* and *B*. These results corroborate the MCA-based findings on role of glucocorticoid receptor in metabolic dysfunction.

### Causal Inference

**Fig. 7.**
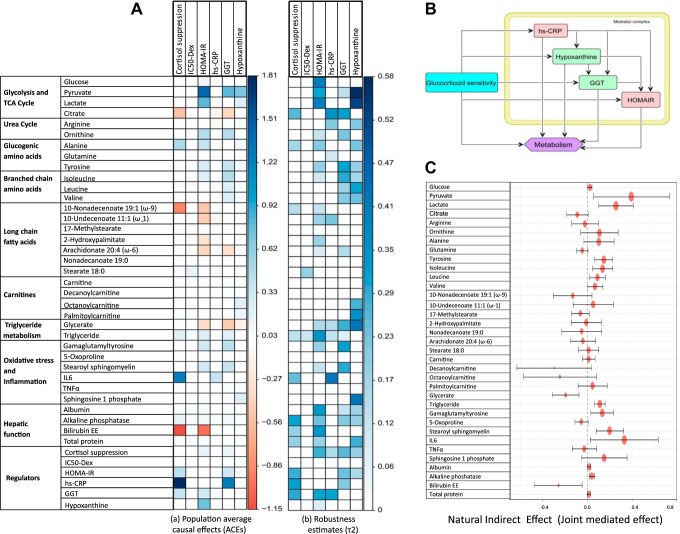
*A*: plots for average causal estimates (ACEs) for regulatory components on metabolites (*left*). *Right* shows the robustness estimates obtained through sensitivity analysis. ACEs are shown for the statistically significant effects (*P* < 0.05; Supplemental Table S7). Cortisol suppression (CS) shows negative effect on citrate, bilirubin, and nonadecanoate but positive effect on triglycerides, IL6, alkaline phosphatase, plasma proteins, homeostatic model assessment of insulin resistance (HOMA-IR), γ-glutamyltransferase (GGT), and high-sensitivity C-reactive protein (hs-CRP). HOMA-IR shows an identical effect to metabolic dysfunction (MD) signature for glycolytic metabolites, amino acids, fatty acids, CS, GGT, and hypoxanthine along with hepatic function. hs-CRP shows a negative association with citrate, glutamine, undecenoate, and glycerate and a positive effect on IL6, CS, triglyceride, and GGT. GGT shows a causal effect identical to the MD signature for pyruvate and citrate, ornithine, amino acids, triglycerides, stearoyl sphingomyelin and γ-glutamyltyrosine, hepatic function components, CS, HOMA-IR, and hs-CRP. Hypoxanthine corroborates with several features of MD signature: pyruvate, lactate, amino acids, carnitines, and HOMA-IR along with glycerate, sphingosine 1 phosphate, hepatic function, and CS. *B*: causal graph used to test for mediation hypothesis of the effect of GRS measured by dexamethasone (Dex) suppression test (DST). Mediator complex of hs-CRP, hypoxanthine, GGT, and HOMA-IR was considered as the joint mediators. *C*: forest plot representation of the natural indirect effects (joint-mediated effects) of increased GC feedback sensitivity (measured by cortisol suppression test) on 35 metabolites for the causal hypothesis tested on the entire cohort adjusting for the group effects (Supplemental Table S8). Error bar represents 95% confidence intervals of the point estimates of the effects. It is noted that the joint-mediated effects on pyruvate, lactate, citrate, gluconeogenic and branched-chain amino acids, oxidative stress, inflammation, and hepatic function components are statistically significant.

To assess the model-based hypothesis on the effects of variables associated with pathways inferred from MCA, we performed causal inference between the regulatory components and the metabolites that show statistically significant differences in the two cohorts. Since our data come from an observational study, we used covariate balancing propensity scores (CBPS) for weighting in estimation of average causal effects (16) and performed sensitivity analysis to determine the extent of violation of the ignorability assumption (20). Since we focused on analyzing the effect of GC receptor sensitivity, we tested the causal hypothesis for 6 regulatory components [dexamethasone cortisol suppression (CS), IC_50_, HOMA-IR, hs-CRP, GGT, and hypoxanthine] with respect to 35 features by estimating and testing the sensitivity of average causal effects (ACEs: γ) to unmeasured confounding. We used 8 covariates, namely, age, body mass index, education, race, ethnicity, current medications (10 categories: antidepressants, sedatives, anticonvulsants, antidiabetics, antiallergic, anti-inflammatory, antihypertensive, pain medicine, antacid, and statins), and smoking and alcohol use, to ensure the covariate balance on the exposure variable. ACEs along with their statistics and robustness estimate (τ_1_ and τ_2_) are reported in Supplemental Table S7. Figure 7*A* shows the statistically significant ACEs and the corresponding robustness estimates for the 6 regulatory components.

We observed that cortisol suppression showed strong causal association with multiple regulatory components, such as HOMA-IR, hs-CRP, and GGT, and these regulatory components were further causally associated with hypoxanthine and several metabolic pathways. This indicated the possibility of mediation of the effects of changes in GC sensitivity on metabolites through these components. To validate this, we next performed causal mediation analysis on the causal structure as shown in Fig. 7*B*. To obtain CS-specific effects, we also controlled for urinary catecholamine to account for the effects of epinephrine on the β-adrenergic pathway, which could also yield the MD signature as observed by the MCA, and dehydroepiandrosterone due to its effect on HPA axis and insulin sensitivity. For the sake of simplicity, we assume an acyclic graph to identify the causal effects. Because of possible bidirectionality of the effects within the regulatory components, we evaluated joint mediated effects through the mediator complex to satisfy ignorability. The detailed reporting of the summary statistics can be found in Supplemental Table S8. Figure 7*C* shows the estimates with 95% confidence interval for natural indirect effect (joint mediated effects) by cortisol suppression (GR sensitivity) on the metabolites. Refer to Supplemental Table S8 and Supplemental Fig. S2 for significant mediated effects, natural direct effect (NDE), and total causal effects.

## DISCUSSION

### Mechanistic Inference from MCA and Causal Analysis

We reconcile the findings from MCA and associated regulatory states to mechanistically explain the effect of trauma-induced changes in HPA axis on metabolism. We then consolidate the explanations with the findings from our data. The regulatory landscape observed in model simulations for the parameter perturbations (Supplemental Table S5 and Fig. 5) indicates that changes in metabolism along with increased GR sensitivity and inflammation are mechanistically associated with energy deficit. At the physiological level, how the processes identified by the MCA (GR sensitization, inflammation, insulin resistance, and GPCR activation) can result in an energy deficit and the metabolic phenotypes observed in our PTSD sample (Fig. 2) are discussed below in light of the causal analysis and supporting literature.

#### Increased insulin resistance.

Confronted with exposure to a traumatic event, stress hormones (cortisol and catecholamine) are known to initiate gluconeogenesis to facilitate hepatic glucose production to cope with the energy requirements of an excessive activity in brain and muscles (64). These metabolic shifts are achieved by reducing insulin sensitivity and increasing the β-adrenergic receptor signaling (acute stress) and glucocorticoid receptor signaling (containment of acute stress response) to enhance catabolism for glucose production and use. The effect of insulin insensitivity is further compensated by elevation of plasma insulin secretion to restore plasma glucose levels. In our data, this is observed as insulin resistance indicated by increased HOMA-IR (*q* = 0.007) and a significant ACE of cortisol suppression on HOMA-IR (γ = 0.411, *P* = 0.003, *q* = 0.016, τ_2_ = 0.291). Moreover, in insulin-dependent tissues (muscle and adipose), insulin action is required for glucose transport and mitochondrial conversion of pyruvate to acetyl-CoA; hence, insulin resistance would affect efficient ATP generation. Our data show a significant association of hypoxanthine with HOMA-IR (ρ = 0.292, *P* = 1.44E−4, *q* = 0.001) corroborating plausibility of this mechanism.

#### Increased gluconeogenesis.

Induction of gluconeogenesis in liver is associated with increase in pyruvate derived from gluconeogenic precursors such as amino acids (alanine, glycine, cysteine, and serine; Ref. 37). Gluconeogenesis consumes an equivalent of 11 ATPs for driving pyruvate to glucose with a loss of 8 ATPs that could be generated if pyruvate had been processed in mitochondria. Therefore, it is an energy-consuming process, and its sustained upregulation would lead to an energy-deficient state with changes in ATP-to-ADP and NADH-to-NAD ratios, thereby affecting all of the pathways that depend on adenosine phosphorylation and redox potential for their substrate utilization. Our data show a significant association of cortisol suppression with glucose (ρ = 0.214, *P* = 0.006, *q* = 0.02) and hypoxanthine with glucose (ρ = 0.217, *P* = 0.005, *q* = 0.019) corroborating this mechanism.

#### Potential hypoxic adaptation.

In the liver, increased diversion of pyruvate to gluconeogenesis can reduce mitochondrial substrate availability, resulting in reduced levels of citrate (*P* = 0.039) and subsequent α-ketoglutarate (αKG). It is known that HIF1α is sensitive to the changes in the level of αKG; therefore, reduced αKG would induce upregulation of HIF1α (18). Additionally, the upregulation of proinflammatory state is known to induce hypoxia with upregulation of HIF1α (3). Although HIF1α would act to sensitize insulin action (83), the concomitant inflammation would maintain insulin resistance and subsequent gluconeogenesis in liver.

In the tissues like muscle that use glucose, upregulation of HIF1α is known to influence glycolytic enzymes, leading to lactate accumulation, which in turn stabilizes HIF1α (38). This leads to reduced TCA flux and lower ATP production through oxidative phosphorylation. Therefore, to compensate for the lower supply of phosphate groups in metabolic reactions, an increased breakdown of ADP to AMP leads to activation of AMP kinase that upregulates glycolysis (79). These overall may lead to aerobic glycolysis and an energy-deficient state. The plausibility of such a mechanism is supported by our data with the significant ACE of hypoxanthine on pyruvate (γ = 0.695, *P* = 1.07E−12, *q* = 3.29E−11, τ_2_ = 0.583) and lactate (γ = 0.248, *P* = 3.19E−13, *q* = 1.12E−11, τ_2_ = 0.532). We also observed a significant association of cortisol and cortisol suppression with pyruvate and lactate (see Supplemental Table S6) and a negative ACE of cortisol suppression on citrate (γ = −0.534, *P* = 0.005, *q* = 0.026, τ_2_ = 0.259). Moreover, hypoxanthine showed a statistical trend of negative association with plasma phosphate levels (ρ = −0.23, *P* = 0.04) and a positive association with AMP (ρ = 0.21, *P* = 0.06) in our subjects with PTSD.

#### Reduced β-oxidation of fatty acids.

During catabolic state, it is crucial to maintain the cellular levels of NADH and ATP; therefore, gluconeogenesis is usually associated with fatty acid β-oxidation to maintain the steady supply of NADH and ATP. The gain of ATP from β-oxidation is relatively high, such as the ATP yield from oxidation of a typical palmitoyl-CoA is ~106 ATP accounting for 2 ATPs used in fatty acid activation. Although the conventional catabolic signaling would upregulate fatty acid β-oxidation, the sustained higher levels of GCs or active GR signaling are known to inhibit β-oxidation in hepatocytes (41). This is also indicated in our data from a significant association between plasma cortisol levels and carnitine derivatives (see Supplemental Table S6*A*). Moreover, upregulation of HIF1α (hypoxia) is shown to be associated with reduction in β-oxidation (46) limiting carnitine utilization, which is also corroborated in our data by the significant ACE of hypoxanthine on carnitine derivatives (palmitoylcarnitine: γ = 0.252, *P* = 2.45E−7, *q* = 5.02E−6, τ_2_ = 0.576). This action would reduce mitochondrial carnitine utilization and further reduce ATP availability from β-oxidation, indicating another mechanism for energy deficit. Our subjects with PTSD and another study on both animals and humans with PTSD (90) showed increased levels of carnitines, indicating the possibility of this mechanism.

#### Impaired lipid metabolism.

Although glucocorticoids induce insulin resistance (IR) in liver and muscle, they are known to sensitize insulin signaling in adipose tissues (26). Therefore, hyperinsulinemia due to IR would sensitize adipose for triglyceride synthesis, which is also associated with increased GC activity (62). Triglyceride synthesis is an energy-intensive process that consumes six ATPs per three molecules of fatty acids and one molecule of glycerol. Therefore, the sustained higher activity of GCs would further add to the effect of lowering ATP availability. This action can be corroborated from our data by significant positive ACE of HOMA-IR (γ = 0.235, *P* = 5.31E−6, *q* = 8.71E−5, τ_2_ = 0.34) and cortisol suppression (γ = 0.213, *P* = 0.017, *q* = 0.07, τ_2_ = 0.135) on plasma triglyceride levels.

Fatty acid synthesis is a redox-dependent process that consumes ATP and NADH and is regulated by insulin action (19). The reduction in mitochondrial citrate (*P* = 0.039) due to increased lactate flux can lead to reduced substrate availability for lipogenesis. Therefore, the lower availability of these substrates and insulin resistance can reduce lipogenesis in nonadipose tissues, whereas high GCs drive fatty acids to triglyceride synthesis in adipose tissues. These dual actions can lead to lower levels of circulating fatty acid. This is indicated by significant negative causal association of HOMA-IR and cortisol suppression with fatty acids in our data (see Supplemental Tables S6*A* and S7).

#### Impaired amino acid metabolism.

Proteins may be broken down to produce amino acids, ready to be converted into usable molecules for gluconeogenesis (or the TCA cycle to produce energy) through anaplerosis. Whereas alanine (*P* = 0.015) is directly converted to pyruvate, glutamine (*P* = 0.021) and tyrosine (*P* = 0.005) enter the TCA cycle at αKG and fumarate nodes, respectively. Stress-induced GC activity is known to upregulate proteolysis (9). Similarly, our data show a significant ACE of cortisol suppression on total plasma proteins (γ = 0.056, *P* = 0.014, *q* = 0.058, τ_2_ = 0.198) and a trend-level effect on alanine and tyrosine. Efficient insulin action is essential for inhibition of proteolysis (17); therefore, insulin resistance would enhance the proteolytic effect adding to amino acid pool in the plasma. This is indicated by significant causal association between HOMA-IR and amino acids (see Supplemental Table S7) in our data. Under tonic hypoxia, HIF1α is also known to upregulate glutaminolysis (78) in an attempt to restore mitochondrial redox potential, which is also indicated in our sample by negative association between hypoxanthine and glutamine (ρ = −0.159, *P* = 0.041, *q* = 0.098). Moreover, an efficient catabolism of branched-chain amino acids (BCCAs) contributes to the pool of NADH and ATP generation in muscle; therefore, impaired BCCA catabolism would lead to lower energy supply and increased BCCAs in the circulation as observed by significant associations between BCCAs, GGT, and hypoxanthine in our data (see Supplemental Table S7). Furthermore, amino acids have an ability to influence glucagon secretion and induce gluconeogenesis (10), leading to an autopositive feedback effect on energy deficit.

#### Impaired urea cycle and NO production.

The processing of amino acids involves deamination generating ammonia that needs to be purged from the body through the urea cycle (82). In the urea cycle, formation of carbamoyl phosphate from ammonia and carbon dioxide requires two ATPs. The reduced availability of ATP can thus limit formation of carbamoyl phosphate and its subsequent reaction with ornithine, thereby leading to accumulation of ornithine (*P* = 0.01) and reduced levels of citrulline and arginine (trend) as observed in our data. Furthermore, elevated arginase expression is shown to be associated with increased inflammatory cytokines and GC activity (59), which could further lead to a lower urea cycle flux, reduced arginine levels, and subsequent nitric oxide production (5). This is also supported by a significant positive association between cortisol suppression and ornithine (ρ = 0.223, *P* = 0.004, *q* = 0.015) and a trend of causal association between hs-CRP and arginine (γ = −0.05, *P* = 0.05, *q* = 0.145, τ_2_ = 0.211).

#### Increased β-adrenergic GPCR signaling.

The upregulation of β-adrenergic GPCR signaling by catecholaminergic and nongenomic actions of glucocorticoid receptor signaling (29) is known to activate cAMP and calcium signaling that influence TCA enzymes and oxidative phosphorylation for production of NADH and ATP, respectively. However, sustained elevation in the cellular calcium signal stimulates higher mitochondrial reactive oxygen species (ROS) generation (28) due to enhanced electron flow through respiratory chain and subsequent inhibition of complexes I and III of ubiquinone (Q) cycle. ATP depletion and increase in AMP-to-ATP ratios are also associated with increase in ROS generation (53). In our sample, urinary epinephrine (activator of β-adrenergic signaling) showed a significant correlation with hypoxanthine (ρ = 0.17, *P* = 0.029, *q* = 0.07), implying an additional role of enhanced GPCR signaling in energy-deficient state.

#### Increased GR sensitivity and inflammation.

Although glucocorticoids are widely known for their anti-inflammatory activity, the proinflammatory effects are also documented (22). Our model analysis showed that increasing GR sensitivity of central negative feedback of HPA axis and its anti-inflammatory activity increased the proinflammatory response. This is because of the central negative feedback loop that leads to reduction in the gain on ACTH-stimulated cortisol release and subsequent reduction in the anti-inflammatory response (70). This results in increased proinflammatory milieu that acts to further upregulate HPA axis in an attempt to restore homeostasis, however, at the cost of dysregulated immune response (55). Our sample revealed a significant and robust positive causal association of cortisol suppression with hs-CRP (γ = 1.808, *P* = 0.009, *q* = 0.038, τ_2_ = 0.315) and IL6 (γ = 1.234, *P* = 0.026, *q* = 0.091, τ_2_ = 0.255) and a trend-level reduction in IC_50_ levels (*P* = 0.08). These indicate the plausibility of such a mechanism for increased GR sensitivity of central negative feedback and inflammatory response.

#### Interplay of oxidative stress, inflammation, insulin resistance, and energy deficit.

Chronic exposure to higher glucocorticoid is associated with oxidative stress (6). Oxidative stress is known to induce insulin resistance (31), inflammation (14), and activation of HIF1α. Systemic inflammation is known to induce insulin resistance (15) and oxidative stress. Moreover, inflammatory cytokines and ROS are known to activate sphingosine 1 phosphate and are implicated in pathophysiology of hypoxia and psychiatric disorders (49, 52). These interactions are also supported by our data with significant ACEs among HOMA-IR, GGT, hs-CRP, and hypoxanthine (see Supplemental Tables S6*A* and S7). Corroborating in our data, we also observed the correlates of impaired antioxidant pathway noted by significant differences in γ-glutamyltyrosine (*P* = 0.008) and oxoproline (*P* = 0.002) and the elevated levels of stearoyl sphingomyelin (*P* = 0.006) that are implicated in oxidative stress, insulin resistance, and inflammation (1a). We also observed significant causal associations of sphingosine 1 phosphate with hypoxanthine (γ = 0.252, *q* = 5.02E−6, τ_2_ = 0.428) suggesting an association of inflammation and energy deficit. Moreover, IC_50_-DEX showed a significant correlation with hypoxanthine indicating an association of immune GR sensitivity with hypoxia (40). These multiple interactions between comorbidities would constitute a mechanism for positive feedback on mitochondrial dysfunction.

#### Effects of sensitive GR on metabolism are jointly mediated by regulatory complex.

Through the model simulations, for a modest increase in GR sensitivity, we observed increase in inflammatory cytokines, plasma insulin, and glucose indicating insulin resistance, hypoxic response, and impaired NADH-to-NAD and ATP-to-ADP ratios indicating energy deficit. These features were collectively responsible for the metabolic phenotype observed in the MD signature. The model-based observations matched well with the interplay of these phenotypes in our data as described in earlier sections. Accordingly, we observed a significant ACE of cortisol suppression on hs-CRP (γ = 1.394, *q* = 0.067, τ_2_ = 0.207), HOMA-IR (γ = 0.372, *q* = 0.01, τ_2_ = 0.265), and GGT (γ = 0.32, *q* = 0.028, τ_2_ = 0.256). Moreover, the causal mediation analysis showed that the effect of increased GR sensitivity on metabolic phenotypes of abnormalities in glycolysis, TCA cycle, amino acid metabolism, triglyceride metabolism, and hepatic function were jointly mediated by these regulatory components along with energy deficit (hypoxanthine; Supplemental Table S8).

### Strengths and Limitations

Our analysis involves a systems approach to identify mechanisms that may cause metabolic dysfunction consistent with group differences in our cohort. We made a novel attempt to integrate earlier observations at multiple levels of the HPA axis, immune system, metabolism, and their regulations together in a form of a systems model. This multilevel analysis provides insight on etiological aspects of physiological response to chronic stress and the resulting metabolic dysfunction. We shed light on the mechanistic understanding of how the defects in GR signaling might affect systemic metabolic regulation yielding the state of energy deficit and concomitant changes in metabolic landscape as observed in our subjects with PTSD. The mechanistic insights obtained from our analysis can be generalized to glucocorticoid-related diseases that involve metabolic dysfunction.

From the model analysis, our inferences are limited by the modeling assumptions and what was observed by model perturbations predominantly in hepatic metabolism; however, the systemic effects may produce varied results. Because of the lack of availability of quantitative data for several subsystem variables in the same cohort, we have extensively relied on the previous literature for the data, and the parameter estimation was performed to fit the qualitative profiles under different physiological conditions using a semiempirical approach. Hence, alternative parameter fits may be possible for the actual quantitative data and the model may be subject to further improvement. Since the data for all of the variables in the model were not available for a single cohort, we had to depend on literature sources for relevant parameter values.

Not all individuals who are exposed to trauma will develop PTSD; therefore, our design is observational. It would be practically infeasible and unethical to randomize individuals and expose them to trauma or modulate GR sensitivity in people. Hence, to test the model-based hypothesis, we used the results of in vivo dexamethasone suppression test as a proxy for systemic GR sensitivity in our subjects and the continuous exposure variable for causal inference. Since the causal structure assumed was an acyclic graph, it might be subject to weaker assumptions on sequential ignorability.

### Conclusion

In summary, our model suggests that increasing the Hill coefficient (a measure of cooperativity due to receptor dimerization) of GR’s effects sensitizes central negative feedback response in HPA axis and reduces GR’s anti-inflammatory effect at ambient cortisol levels due to relative difference in their GR inhibitory thresholds. Both actions synergistically potentiate GR nuclear translocation, the former due to direct sensitization and the latter due to increased proinflammatory cytokines that positively feedback on the HPA axis. The enhanced GR nuclear action and increased inflammation together affect downstream metabolic regulation and bioenergetics. Hence, we infer that the metabolic phenotype of MD observed in PTSD could at least in part be due to the effect of trauma-induced glucocorticoid sensitivity that may result in inflammation, insulin resistance, oxidative stress, and subsequent energy deficit, which was supported by the correlational and causal analysis using our data. Our findings of the defective energy metabolism also replicated the findings reported by other studies on patients with psychiatric disorders (91) and in PTSD-specific population (24, 77), implying mitochondrial dysfunction in PTSD. Our analysis indicates potential of targeting redox regulation (67), inflammation, and modulation of GR sensitivity as the therapeutic alternatives for managing metabolic abnormalities in PTSD, motivating further controlled experiments to validate the causal role of enhanced GC sensitivity in metabolic dysregulation and test its therapeutic potential for managing metabolic dysfunction in PTSD.

## GRANTS

This work was supported by a research grant from US Army Medical Research and Materiel Command under Award nos. W911NF-17-2-0086, W81XWH-10-1-0021 (principal investigator: O. M. Wolkowitz), and W911NF-17-1-0069 (principal investigator: B. J. Daigle).

## DISCLAIMERS

The views, opinions, and findings contained in this report are those of the authors and should not be construed as official Department of the Army position, policy, or decision, unless so designated by other official documentation. Citations of commercial organizations or trade names in this report do not constitute an official Department of the Army endorsement or approval of the products or services of these organizations.

## DISCLOSURES

No conflicts of interest, financial or otherwise, are declared by the authors.

## AUTHOR CONTRIBUTIONS

P.R.S. and F.J.D. conceived and designed research; P.R.S., J.D.F., and D.A.-A. performed experiments; P.R.S. analyzed data; P.R.S. and S.H.M. interpreted results of experiments; P.R.S. prepared figures; P.R.S. drafted manuscript; P.R.S., S.H.M., J.D.F., D.A.-A., P.S.B.C., O.M.W., R.Y., M.J., L.H., and C.M. edited and revised manuscript; P.R.S., S.H.M., J.D.F., D.A.-A., P.S.B.C., O.M.W., R.Y., M.J., L.H., C.M., and F.J.D. approved final version of manuscript.

## APPENDIX: INTEGRATED MODEL DEVELOPMENT

### Modifications of Source Models for Integration

The metabolic network and the signaling and transcriptional regulatory network integrated and used in this study are shown in Fig. 3, *B* and *C*. The metabolic model was updated to include the regulatory effects of glucocorticoids, inflammatory cytokines, and HIF1α on metabolic enzymes in the metabolic network shown in Fig. 3*A*. The previously developed model for the HPA axis by our group was parameterized to fit the data from Ref. 74. The model interaction of inflammatory cytokines with HPA axis was adapted from Bangsgaard et al. (2) with additional cytokine effects on cortisol secretion adapted from the literature (Supplemental Table S3) and modeled accordingly. As we were only interested in steady-state behavior of the models, in the current model analysis, circadian dynamics was ignored. Whereas the model for inflammation was modified to include the effects from insulin and GPCR signaling pathways, the hypoxia signaling model by Nguyen et al. (56) was updated to include the effects from inflammation and metabolism (TCA intermediates) on HIF1α. The transcriptional regulation model from Somvanshi et al. (71) was modified to include the effects from HPA axis and inflammation. The regulatory functions were applied to metabolic pathways with an assumption of parallel activation mechanism (42), wherein, if a rate-limiting step in a pathway is regulated by a regulator, the regulatory effect is applied to a series of reactions in the pathway until a branch point, store, sink, or next regulated variable of the pathway. The additional interactions and their sources implemented in the integrated model are reported in Supplemental Table S3.

### Parameter Estimation

The majority of the model parameters were maintained from the source models. The parameters encoding the regulatory interactions and the integration of models were obtained from the literature as reported in Supplemental Table S3. We used a semiempirical approach for model integration wherein we emphasized on reproducing the qualitative trends for the model state variables under different conditions as reported in corresponding literature. We fitted the Hill functions by minimizing the least-squares error to the trends reported in literature. The principle of parallel activation of pathways by regulatory influence was used while assigning regulatory effects on the metabolic pathways. We used simulated annealing in MATLAB to reparametrize the models of HPA axis and inflammation after inclusion of the regulatory functions in the integrated model. Although the values for fold change were used from the literature, to avoid overfitting, the choices of *K*_m_ values were made based on the available data, information on the sensitivity of an interaction and the fold variation observed for a regulatory state. Wherever data were not available, a reasonable estimate of *K*_m_ values was made in terms of folds of the basal values of the states as reported in the literature. The time units for the rate parameters in our metabolic model are in minutes, and the concentrations of the metabolites are reported in millimoles unless reported differently in the source models. The time scale differences (minutes and hours) in the submodels were scaled in the integrated model for the simulations. In Supplemental Table S3, we report the values of thresholds (*K*_m_ or *K*_i_) based on the estimates expressed in terms of the folds of the basal values for transcriptional and signaling effects and sensitivity (*n*) assumed according to the steepness of the input-output response. The *V*_max_ represents the fitted estimates required to impart the observed fold change effect in the corresponding data. These rate functions are further scaled in the integrated model to obtain the resultant rates in the ODEs such that the overall state response in the model simulations matches the output of the source models. The parameters for HPA-immune axis and transcriptional regulation are reported in Supplemental Table S4.

### Qualitative Validation of Model

Since our goal was to use the model for hypothesis generation and perform a “what if” kind of analysis, we were interested in a model that could reasonably reproduce the qualitative trends in the observed physiology. We mostly rely on the published models for the model performance and retain the qualitative features of the model in our integrated model after incorporation of additional interactions reported in literature. Because of the large numbers of states, we relied on the source models for the validation. However, the model was qualitatively validated for the reported trends and observations of several experimental reports on the output response with respect to model inputs of the source models such as response to meal input for metabolic model, stress response of the HPA axis, and response to LPS for the inflammation model. Supplemental Fig. S1 shows the qualitative profiles for the model simulations for meal response, stress response, and the response for infection induced by LPS impulse. The trends observed in the figure match the qualitative and quantitative features of the source models and the observed physiological responses in the respective cases.

### Model Equations for Integrated HPA Axis-Inflammation Model

Below, we report only the integrated model of HPA axis, inflammation, and the transcriptional compartments. The ODE model for the metabolic simulator can be found at https://github.com/pramodrajarams/metabolic-model.

Corticotropin-releasing hormone (CRH):

$$\overset{˙}{\text{Crh}}=K_{\text{strs}}\times \left(\frac{{K\text{i}}_{n1}^{n}}{K{\text{i}}_{n1}^{n}+\text{GR}n^{n}}\right)+q3\times \left(\frac{\text{TNF}}{q8+\text{TNF}}\right)-Vs3\times \text{Crh}$$

Adrenocorticotropic hormone (ACTH):

$$\dot{\text{Acth}}=K_{\text{p}}2\times \text{Crh}\times \left(\frac{K{\text{i}}_{n1}^{n}}{K{\text{i}}_{n1}^{n}+\text{GR}n^{n}}\right)\times \left[1+q4\times \left(\frac{{\text{TNF}}^{n3}}{q5^{n3}+{\text{TNF}}^{n3}}\right)\right]-Vs4\times \text{Acth}$$

Plasma cortisol:

$$\dot{\text{Cort}}=K\text{p}4\times \text{Acth}\times \left\{1+\left[\frac{\text{TNF}}{1+q6\times \left(\text{TGF}+\text{IL}6\right)}\right]\right\}-Vs5\times \text{Cort}$$

Interstitial cortisol:

$$\dot{\text{Cort}p}=\left(\frac{1}{\text{τ}p}\right)\times \left(\text{Cort}-\text{Cort}p\right)$$

Glucocorticoid receptor mRNA:

$$\dot{\text{GRmrna}}=K{\text{synt}}_{\text{rm}}\times \left[1-\left(\frac{\text{GR}n^{nx}}{K{\text{m}}_{\text{Gr}n}^{nx}+\text{GR}n^{nx}}\right)\right]+v\text{tnf}\times \left(\frac{{\text{TNF}}^{m}}{{\text{TNF}}^{m}+K{\text{p}}_{\text{tnf}}^{m}}\right)-K{\text{deg}}_{\text{rm}}\times \text{GRmrna}$$

Glucocorticoid receptor protein:

$$\dot{\text{GRprt}}=K{\text{synt}}_{\text{rp}}\times \text{GRmrna}+vp_{\text{rp}}\times \text{GR}n-K\text{on}\times \text{Cort}p\times \text{GRprt}-K{\text{deg}}_{\text{rp}}\times \text{GRprt}$$

Cytosolic glucocorticoid receptor:

$$\dot{\text{GR}f}=K\text{on}\times \text{Cort}p\times \text{GRprt}-K\text{rt}\times \text{GR}f$$

Nuclear glucocorticoid receptor:

$$\dot{\text{GR}n}=K\text{rt}\times \text{GR}f-K\text{re}\times \text{GR}n$$

$$\text{PI}3{\text{K}}_{\text{eff}}=wt\times \left\{1+\left[f\text{pi}3\text{k}\times \left(\frac{\text{PI}3{\text{k}}^{n4}}{\text{PI}3{\text{k}}^{n4}+K\text{mpi}3{\text{k}}^{n4}}\right)\right]\right\}$$

$${\text{Creb}}_{\text{eff}}=f\text{creb}\times \left(\frac{{\text{CREB}}^{n4}}{{\text{CREB}}^{n4}+K\text{m}{\text{creb}}^{n4}}\right)$$

$${\text{mTOR}}_{\text{eff}}=wt\times \left[1+f\text{mtor}\times \left(\frac{{\text{mTOR}}_{\text{Raptor}}^{n4}}{K{\text{mtor}}^{n4}+{\text{mTOR}}_{\text{Raptor}}^{n4}}\right)\right]$$

Lipopolysaccharide (LPS):

$$\dot{\text{LPS}}=It-dlp\times \text{LPS}\times \text{Phg}$$

Phagocytes:

$$\dot{\text{Phg}}=K\text{phg}\times \left\{\left[1+K\text{ptnf}\times \left({\text{mTOR}}_{\text{eff}}\right)\times \left(\frac{\text{TNF}}{xn\text{TNF}+\text{TNF}}\right)\right]\times \left[\frac{x\text{TGF}}{x\text{TGF}+\text{TGF}}\right]\times \left[\frac{x\text{IL}10}{x\text{IL}10+\text{IL}10}\right]\right\}\times \text{LPS}-dpg\times \text{Phg}$$

Transforming growth factor (TGF):

$$\dot{\text{TGF}}=K\text{tgf}\times \text{Phg}\times \left(\text{PI}3{\text{K}}_{\text{eff}}\right)+\left[q1\times \frac{\text{GR}n^{nx}}{{\left(q2\right)}^{nx}+\text{GR}n^{nx}}\right]-d\text{tgf}\times \text{TGF}$$

Tumor necrosis factor (TNF):

$$K\text{TGF}=x\text{tntg}\times wt\times \left(1+{\text{Creb}}_{\text{eff}}\right)$$

$$\dot{\text{TNF}}=\left(\frac{\text{Phg}}{K\text{TNF}n+\text{Phg}}\right)\times \left(\frac{K{\text{TGF}}^{nf1}}{K{\text{TGF}}^{nf1}+{\text{TGF}}^{nf1}}\right)\times \left\{K\text{tnf}+\left[K\text{tnf}f\times \frac{\text{TNF}}{x\text{TNF}+\text{TNF}}\right]\right\}-d\text{tnf}\times {\text{TNF}}^{2}$$

Interleukin-10 (IL10):

$$\dot{\text{IL}10}=s\text{il}+\left(K\text{IL}10\times \frac{{\text{Phg}}^{nf4}}{x{\text{il}}^{nf4}+{\text{Phg}}^{nf4}}\right)+\left(K\text{iltg}\times \frac{{\text{TGF}}^{nf2}}{x{\text{iltg}}^{nf2}+{\text{TGF}}^{nf2}}\right)-d\text{il}\times \text{IL}10$$

Interleukin-6 (IL6):

$$K\text{IL}10=x\text{il}6\text{il}10\times wt\times \left(1+{\text{Creb}}_{\text{eff}}\right)$$

$$\dot{\text{IL}6}=\text{il}6\text{b}+K\text{il}6\times \left(\frac{{\text{Phg}}^{nf3}}{x\text{il}6^{nf3}+{\text{Phg}}^{nf3}}\right)\times \left[1+K\text{il}6\text{tnf}\times \left(\frac{\text{TNF}+\text{IL}6}{x\text{il}6\text{tnf}+\text{TNF}+\text{IL}6}\right)\right]\times \left(\frac{K\text{IL}10}{K\text{IL}10+\text{IL}10}\right)-d\text{il}6\times \text{IL}6$$

### Model Equations for Transcriptional Regulatory Network

Sterol regulatory element-binding proteins (SREBP):

$${\text{GCR}}_{Ptv}=f\text{gcr}\times \left(\frac{\text{GR}n^{s2}}{K\text{m}{\text{gcr}}^{s2}+\text{GR}n^{s2}}\right)$$

$$Inseff=\frac{\text{AKT}+\text{PKC}}{{\text{AKT}}_{b}+{\text{PKC}}_{b}}$$

$$\text{S}6{\text{k}}_{Ptv}=f\text{s}6\text{k}\times \left(\frac{\text{S}6\text{K}1{\text{p}}^{s2}}{\text{S}6\text{K}1{\text{p}}^{s2}+K\text{ms}6{\text{k}}^{s2}}\right)$$

$${\text{cAMP}}_{Ntv}=f\text{cAMP}\times \left(\frac{K\text{i}{\text{cAMP}}^{s2}}{K\text{i}{\text{cAMP}}^{s2}+{\text{cAMP}}^{s2}}\right)$$

$${\text{AMPK}}_{Ntv}=f\text{AMPK}\times \left(\frac{K\text{i}{\text{AMPK}}^{s2}}{K\text{i}{\text{AMPK}}^{s2}+{\text{AMPK}}_{\text{Eff}}^{s2}}\right)$$

$${\text{FOXO}}_{Ntv_{\text{SREBP}}}=\left(\frac{K\text{i}{\text{FOXO}}^{s2}}{{\text{FOXO}}^{s2}+K\text{i}{\text{FOXO}}^{s2}}\right)$$

$${\text{AKT}}_{{\text{PKC}}_{Ptv_{\text{SREBP}}}}=fins\times \left(\frac{Inseff^{s2}}{Inseff^{s2}+Kins^{s2}}\right)$$

$$\dot{\text{SREBP}}=K\text{sr}0\times \text{S}6{\text{k}}_{Ptv}+\left(K\text{sr}1\times {\text{cAMP}}_{Ntv}\times {\text{AMPK}}_{Ntv}\times {\text{FOXO}}_{Ntv_{\text{SREBP}}}\times {\text{AKT}}_{{\text{PKC}}_{Ptv_{\text{SREBP}}}}\right)-K\text{sr}2\times \text{SREBP}$$

Peroxisome proliferator-activated receptor-γ (PPARγ):

$${\text{FFA}}_{Ptv}=f\text{FFA}\times \left(\frac{{\text{FFA}}^{s3}}{{\text{FFA}}^{s3}+K\text{m}{\text{ffa}}^{s3}}\right)$$

$${\text{AKT}}_{Ptv_{\text{PPARγ}}}=f\text{akt}1\times \left(\frac{{\text{Akt}}^{s2}}{{\text{Akt}}^{s2}+K\text{makt}1^{2s2}}\right)$$

$${\text{FOXO}}_{Ntv_{\text{PPARγ}}}=f\text{Foxo}\times \left(\frac{K\text{i}{\text{FOXO}}^{s2}}{{\text{FOXO}}^{2s2}+K\text{i}{\text{FOXO}}^{s2}}\right)$$

$${\text{HIF}}_{Ntv_{\text{PPARγ}}}=\left(\frac{K\text{m}{\text{hif}}^{s2}}{K\text{m}{\text{hif}}^{s2}+{\text{HIF1α}}^{s2}}\right)$$

$$\overset{˙}{\text{PPARγ}}=Kprg0\times \text{S}6{\text{k}}_{Ptv}+\left(Kprg1\times {\text{AKT}}_{Ptv_{\text{PPARγ}}}\times {\text{FOXO}}_{Ntv_{\text{PPARγ}}}\times {\text{HIF}}_{Ntv_{\text{PPARγ}}}\right)+\left(Kprg3\times {\text{FFA}}_{Ptv}\right)-Kprg2\times \text{PPARγ}$$

Peroxisome proliferator-activated receptor-α (PPARα):

$${\text{PKA}}_{Ptv_{\text{PPARα}}}=f\text{pka}\times \left(\frac{{\text{PKA}}^{s2}}{{\text{PKA}}^{s2}+K\text{m}{\text{pka}}^{s2}}\right)$$

$${\text{FFA}}_{Ptv}=f\text{FFA}1\times \left(\frac{{\text{FFA}}^{s2}}{{\text{FFA}}^{s2}+K\text{mffa}1^{s2}}\right)$$

$${\text{PGC}}_{Ptv_{\text{PPARαβ}}}=f\text{pgc}\times \left(\frac{\text{PGC}1^{s2}}{\text{PGC}1^{s2}+K\text{m}{\text{pgc}}^{s2}}\right)$$

$${\text{HIF}}_{Ntv_{\text{PPARαβ}}}=\left(\frac{K\text{m}{\text{hif}}^{s2}}{K\text{m}{\text{hif}}^{s2}+{\text{HIF1α}}^{s2}}\right)$$

$$\dot{\text{PPARαβ}}=Kpra0+\left[Kpra1\times \left({\text{PKA}}_{Ptv_{\text{PPARα}}}+{\text{PGC}}_{Ptv_{\text{PPARαβ}}}\right)\times {\text{HIF}}_{Ntv_{\text{PPARαβ}}}\right]+Kpra3\times {\text{FFA}}_{Ptv}-Kpra2\times \text{PPARαβ}$$

cAMP response element-binding (CREB) protein:

$${\text{PKA}}_{Ptv_{\text{CREB}}}=f\text{pka}2\times \left(\frac{{\text{PKA}}^{s2}}{{\text{PKA}}^{s2}+K\text{mpka}2^{s2}}\right)$$

$${\text{AKT}}_{Ntv_{\text{CREB}}}=f\text{akt}2\times \left(\frac{\text{Akt}}{K\text{makt}2+\text{Akt}}\right)$$

$$\dot{\text{CREB}}=\left[K\text{cr}1\times \left({\text{PKA}}_{Ptv_{\text{CREB}}}+{\text{GCR}}_{Ptv}\right)\times \left(\text{CREB}t-\text{CREB}\right)\right]-K\text{cr}2\times \left(\text{CREB}\times {\text{AKT}}_{Ntv_{\text{CREB}}}\right)$$

Carbohydrate response element-binding protein (ChREBP):

$${\text{Glu}}_{Ptv}=1+f\text{glu}\times \left(\frac{{\text{Glu}}^{5}}{{\text{Glu}}^{5}+K\text{m}{\text{glu}}^{5}}\right)\text{;}\text{PKA}=\frac{\text{PKA}}{{\text{PKA}}_{b}}$$

$${\text{PKA}}_{Ntv}=f\text{pka}3\times \left(\frac{{\text{PKA}}^{s2}}{K\text{mpka}3^{s2}+{\text{PKA}}^{s2}}\right)$$

$${\text{AMPK}}_{Ntv}=f\text{ampk}1\times \left(\frac{K\text{i}{\text{ampk}}^{s2}}{K\text{i}{\text{ampk}}^{s2}+{\text{AMPK}}_{Eff}^{s2}}\right)$$

$$\dot{\text{CHREB}p}=Khr0+\left(Khr1\times {\text{Glu}}_{Ptv}\times {\text{AMPK}}_{Ntv}\right)-Khr2\times \left(\text{CHREB}p\times {\text{PKA}}_{Ntv}\right)$$

Forkhead box protein O1 (FOXO):

$${\text{GlNAc}}_{Ptv_{\text{FOXO}}}=f\text{glnac}\times \left(\frac{{\text{GlNAc}}^{s2}}{{\text{GlNAc}}^{s2}+K\text{m}{\text{glnac}}^{s2}}\right)$$

$${\text{AMPK}}_{Ntv}=f\text{ampk}1\times \left(\frac{K\text{i}{\text{ampk}}^{s2}}{K\text{i}{\text{ampk}}^{s2}+{\text{AMPK}}_{Eff}^{s2}}\right)$$

$${\text{AKT}}_{Ntv_{\text{FOXO}}}=f\text{akt}3\times \left(\frac{\text{Akt}}{\text{Akt}+K\text{makt}3}\right)$$

$${\text{PPARγ}}_{Ntv_{\text{FOXO}}}=f\text{pparγ}\times \left(\frac{K\text{m}{\text{pparγ}}^{s2}}{{\text{PPARγ}}^{s2}+K\text{m}{\text{pparγ}}^{s2}}\right)$$

$$\overset{˙}{\text{FOXO}}=K\text{fr}0+\left[K\text{fr}11\times {\text{AMPK}}_{Ntv}\times {\text{PPARγ}}_{Ntv_{\text{FOXO}}}\times \left({\text{GlNAc}}_{Ptv_{\text{FOXO}}}+{\text{GCR}}_{Ptv}\right)\right]-K\text{fr}2\times \left(\text{FOXO}\times {\text{AKT}}_{Ntv_{\text{FOXO}}}\right)$$

AMP-activated protein kinase (AMPK):

$${\text{AMP}}_{{\text{ATP}}_{\text{AMPK}}}=2\times \left[\frac{\frac{\text{AMP}}{\text{ATP}}}{\left(\frac{K_{\text{AMP}}}{K_{\text{ATP}}}\right)+\left(\frac{\text{AMP}}{\text{ATP}}\right)}\right]$$

$${\text{AKT}}_{Ntv_{\text{AMPK}}}=f\text{akt}4\times \left(\frac{{\text{Akt}}^{s2}}{{\text{Akt}}^{s2}+K\text{makt}4^{s2}}\right)$$

$${\text{PKA}}_{Ntv_{\text{AMPK}}}=f\text{pka}4\times \left(\frac{{\text{PKA}}^{s2}}{K\text{mpka}4^{s2}+{\text{PKA}}^{s2}}\right)$$

$$\overset{˙}{\text{AMPK}}=\left[K\text{am}1\times \left({\text{AMP}}_{{\text{ATP}}_{\text{AMPK}}}\right)\times \left(\text{AMPK}t-\text{AMPK}\right)\right]-K\text{am}2\times \left(\text{AMPK}\right)\times \left({\text{AKT}}_{Ntv_{\text{AMPK}}}+{\text{PKA}}_{Ntv_{\text{AMPK}}}\right)$$

CCAAT/enhancer-binding protein-α (CEBPα):

$${\text{PKC}}_{Ntv_{\text{CEBPa}}}=f\text{pkc}1\times \left(\frac{\text{PKC}}{\text{PKC}+K\text{mpkc}}\right)\text{;}\;\text{cAMP}=\frac{\text{cAMP}}{{\text{cAMP}}_{b}}$$

$${\text{cAMP}}_{Ptv_{\text{CEBPα}}}=f\text{cAMP}1\times \left(\frac{{\text{cAMP}}^{s2}}{{\text{cAMP}}^{s2}+K\text{mcAMP}1^{s2}}\right)$$

$$\dot{\text{CEBPα}}=K\text{cb}0+\left[K\text{cb}1\times \left({\text{cAMP}}_{Ptv_{\text{CEBPα}}}+{\text{GCR}}_{Ptv}\right)\right]-K\text{cb}2\times \left(\text{CEBPα}\times {\text{PKC}}_{Ntv_{\text{CEBPα}}}\right)$$

Peroxisome proliferator-activated receptor-γ coactivator 1α (PGC1α):

$${\text{NAD}}_{Ptv_{\text{PGC}}}=2\times \left(\frac{\frac{\text{NAD}}{\text{NADH}}}{\frac{\text{NAD}}{\text{NADH}}+\frac{K_{\text{NAD}}}{K_{\text{NADH}}}}\right)$$

$${\text{FOXO}}_{Ptv_{\text{PGC}}}=f\text{foxo}1\times \left(\frac{{\text{FOXO}}^{s2}}{{\text{FOXO}}^{s2}+K\text{mfoxo}1^{s2}}\right)$$

$${\text{AKT}}_{Ntv_{\text{PGC}}}=f\text{akt}5\times \left(\frac{\text{Akt}}{\text{Akt}+K\text{makt}5}\right)$$

$${\text{HIF}}_{Ntv_{\text{PGC}}}=\frac{K\text{m}{\text{hif}}^{s2}}{K\text{m}{\text{hif}}^{s2}+{\text{HIF1α}}^{s2}}$$

$${\text{CREB}}_{Ptv_{\text{PGC}}}=f\text{creb}1\times \left(\frac{{\text{CREB}}^{s2}}{{\text{CREB}}^{s2}+K\text{mcreb}1^{s2}}\right)$$

$${\text{AMPK}}_{Ptv_{\text{PGC}}}=f\text{ampk}1\times \left(\frac{{\text{AMPK}}^{s2}}{{\text{AMPK}}^{s2}+K\text{mampk}1^{s2}}\right)$$

$${\text{TNF}}_{Ntv_{\text{PGC}1}}=\left\{1+f\text{tnfil}6\times \left[\frac{{\left(\text{TNF}+\text{IL}6\right)}^{s2}}{K\text{mtnfil}6^{s2}+{\left(\text{TNF}+\text{IL}6\right)}^{s2}}\right]\right\}$$

$$\dot{\text{PGC}1}=K\text{pc}0+\left[K\text{pc}1\times \left({\text{CREB}}_{Ptv_{\text{PGC}}}+{\text{FOXO}}_{Ptv_{\text{PGC}}}+{\text{GCR}}_{Ptv}+{\text{NAD}}_{Ptv_{\text{PGC}}}+{\text{AMPK}}_{Ptv_{\text{PGC}}}\right)\times {\text{HIF}}_{Ntv_{\text{PGC}}}\right]-K\text{pc}2\times \text{PGC}1\times \left({\text{AKT}}_{Ntv_{\text{PGC}}}\times {\text{TNF}}_{Ntv_{\text{PGC}1}}\right)$$

Tribbles homolog 3:

$$\text{PI}3{\text{K}}_{Ptv_{\text{TRB}}}=f\text{pi}3\text{k}\times \left(\frac{\text{PI}3{\text{k}}^{1.5}}{\text{PI}3{\text{k}}^{1.5}+K\text{mpi}3{\text{k}}^{1.5}}\right)$$

$${\text{PKC}}_{Ptv_{\text{TRB}}}=f\text{pkc}2\times \left(\frac{\text{PKC}}{\text{PKC}+K\text{mpkc}2}\right)$$

$${\text{PPAR}}_{Ptv_{\text{TRB}3}}=f\text{ppar}\times \left(\frac{{\text{PPARα}}^{s2}}{{\text{PPARα}}^{s2}+K\text{m}{\text{ppar}}^{s2}}\right)$$

$${\text{PGC}}_{Ptv_{\text{PPARαβ}}}=f\text{pgc}1\times \left(\frac{\text{PGC}1^{s2}}{\text{PGC}1^{s2}+K\text{mpgc}1^{s2}}\right)$$

$$\dot{\text{TRB}3}=K\text{tr}0+\left[K\text{tr}1\times \text{PI}3{\text{K}}_{Ptv_{\text{TRB}}}\times {\text{PKC}}_{Ptv_{\text{TRB}}}\times \left({\text{PPAR}}_{Ptv_{\text{TRB}3}}+{\text{PGC}}_{Ptv_{\text{PPARαβ}}}+{\text{GCR}}_{Ptv}\right)\right]-\left(K\text{tr}2\times \text{TRB}3\right)$$

### Characterization of the Metabolic State

To quantify the net metabolic state (either catabolic or anabolic) per parameter perturbation, we devised an equation analogous to formulation presented for phosphorylation state by Ref. 35. The formulation represents an ensemble of the difference between the scaled effects of state variables of anabolic and catabolic signaling pathways and the corresponding states of transcriptional factors. The metabolic state is scaled between 0 and 1, with a basal state ~0.5. The metabolic state is represented by:

$$\begin{array}{c} \text{Metabolic state}=0.5\times \\ \left(1+\left\{\begin{array}{c} 0.2\times \left[\left(\frac{\text{PKA}}{h_{\text{Pka}}+\text{PKA}}\right)+\left(\frac{\text{Cal}}{h_{\text{Cal}}+\text{Cal}}\right)+\left(\frac{\text{CREB}}{h_{\text{CREB}}+\text{CREB}}\right)+\left(\frac{\text{AMPK}}{h_{\text{AMPK}}+\text{AMPK}}\right)+\left(\frac{\text{HIF1α}}{h_{\text{HIF}}+\text{HIF1α}}\right)\right]\\ -0.2\times \left[\left(\frac{\text{AKT}}{h_{\text{Akt}}+\text{AKT}}\right)+\left(\frac{\text{mTOR}}{h_{\text{mTOR}}+\text{mTOR}}\right)+\left(\frac{\text{SREBP}}{h_{\text{SREBP}}+\text{SREBP}}\right)+\left(\frac{\text{ChREB}p}{h_{\text{ChREBP}}+\text{ChREB}p}\right)+\left(\frac{\text{PPARγ}}{h_{\text{PPARγ}}+\text{PPARγ}}\right)\right] \end{array}\right\}\right) \end{array},$$

where PKA, Cal, CREB, AMPK, and HIF are the catabolic effector state variables and AKT, mTOR, SREBP, ChREBP, and PPARγ are the anabolic state variables. The *h* factor in the denominator is the half-maximal saturation constant that is the scaled difference of the maximum and minimum levels of the corresponding variable.
